# Assessing known chronic kidney disease associated genetic variants in Saudi Arabian populations

**DOI:** 10.1186/s12882-018-0890-9

**Published:** 2018-04-17

**Authors:** Cyril Cyrus, Samir Al-Mueilo, Chittibabu Vatte, Shahanas Chathoth, Yun R. Li, Hatem Qutub, Rudaynah Al Ali, Fahad Al-Muhanna, Matthew B. Lanktree, Khaled Riyad Alkharsah, Abdullah Al-Rubaish, Brian Kim-Mozeleski, Brendan Keating, Amein Al Ali

**Affiliations:** 1 Institute for Research and Medical Consultation, Imam Abdulrahman bin Faisal University, P.O. Box 1982, Dammam, 31441 Saudi Arabia; 2 King Fahd Hospital of the University, Imam Abdulrahman bin Faisal University , Alkhobar, Saudi Arabia; 3 Perelman School of Medicine, University of Pennsylvania, Philadelphia, PA USA; 4 Al-Omran Scientific Chair for hematological diseases, King Faisal University, Al Hassa, Saudi Arabia; 5 Nephrology Division, Department of Medicine, McMaster University, Hamilton, ON L8N 4A6 Canada

**Keywords:** Chronic kidney disease, SNP, MYH9, SHROOM3, Genetic biomarkers, SLC7A9, CST3

## Abstract

**Background:**

Genome wide association studies of patients with European descent have identified common variants associated with risk of reduced estimated glomerular filtration rate (eGFR). A panel of eight variants were selected to evaluate their association and prevalence in a Saudi Arabian patient cohort with chronic kidney disease (CKD).

**Methods:**

Eight genetic variants in four genes (*SHROOM3, MYH9, SLC7A9, and CST3*) were genotyped in 160 CKD patients and 189 ethnicity-matched healthy controls. Genetic variants were tested for association with the development of CKD (eGFR < 60 ml/min/1.73m^2^) and effects were compared with results obtained from 133,413 participants in the CKD genetics consortium. Multivariable regression was used to evaluate the role of these eight variants in improving prediction of CKD development.

**Results:**

All eight variants were present in Saudi populations with minor allele frequency ranging from 16 to 46%. The risk variant in all four genes demonstrated the same direction of effect as observed in European populations. One variant, rs4821480, in *MYH9* was significantly associated with increased risk of development of CKD (OR = 1.69, 95% CI 1.22–2.36, *P = *0.002), but the additional variants were not statistically significant given our modest sample size.

**Conclusions:**

CKD risk variants identified in European populations are present in Saudis. We did not find evidence to suggest heterogeneity of effect size compared to previously published estimates in European populations. Multivariable logistic regression analysis showed a statistically significant improvement in predicting the CKD using models with either FGF23 and vitamin D or FGF23, vitamin D level, and MYH9 genotypes (AUC = 0.93, 95% CI 0.90–0.95, *P* <  0.0001).

**Electronic supplementary material:**

The online version of this article (10.1186/s12882-018-0890-9) contains supplementary material, which is available to authorized users.

## Background

Chronic Kidney disease (CKD) has been rising in prevalence globally in recent years with shifting demographics including an aging population, lifestyle changes associated with urbanization, obesity and type 2 diabetes (T2D). The morbidity and mortality costs of CKD on society are significant, and are projected to become an even larger healthcare burden [1, 2]. In the Kingdom of Saudi Arabia (KSA), CKD is estimated to affect approximately 1.72 million Saudis [3], equating to approximately 6% of the population. Furthermore, in KSA there is a consistent annual increase in the rate of CKD patients undergoing hemodialysis after development of end stage renal disease (ESRD) [4]. In a 2010 KSA investigation only 7.1% of Saudis with CKD were aware of their disease status and such under- and late- diagnoses of CKD often results in poorer outcomes in such patients [3].

A strong heritable component to CKD and its risk factors exists [5, 6], independent of its largest risk factors hypertension and T2D [7, 8]. Monogenic pediatric renal diseases, such as congenital and infantile nephrotic syndromes, appear significantly more common in KSA than in the western world [9]. Over the last decade, genome wide association studies (GWAS) have proven to be an invaluable tool for identifying common variants associated with complex human diseases with small to moderate effects in agnostic hypothesis free approaches [10]. The identified variants and genes can yield novel insights into disease pathogenesis. GWAS of European white populations examining estimated glomerular filtration rate (eGFR) and CKD have identified greater than 50 associated loci [5, 11–14]. Variants in *Shroom Family Member 3* (*SHROOM3)* have been identified as a CKD susceptibility locus through GWAS. SHROOM3 is regulator of epithelial cellular arrangement and planar remodeling [15], which contributes to glomerular filtration barrier integrity [16]. One CKD-associated *SHROOM3* variant, rs17319721, was shown to impact cis-expression and renal allograft fibrosis [17]. Polymorphisms in *Myosin Heavy Chain 9* (*MYH9)* have also been shown to be associated with CKD risk in admixed non-diabetic nephropathy, and focal segmental glomerulosclerosis (FSGS) [18, 19]. Genetic polymorphisms in *Solute Carrier Family 7 Member 9 (SLC7A9)*, an amino acid transporter known to be expressed in renal proximal tubule cells, cause cystinuria [20], are associated with GFR [5, 21], and have been identified as a risk factor for CKD patients of European ancestry [12]. Variants in *Cystatin C* (*CST3)*, have been also shown to impact altered eGFR and kidney disease [22].

While ethnicity is thought to play a large role in CKD genetics, very few genotyping studies of CKD loci have been performed in Saudi patients to date. In this study, we genotyped eight SNPs in four genes which have been shown to be associated with CKD mainly in populations of European descent. Herein we present the allele frequencies of said SNPs in 160 Saudi CKD and 189 non-CKD subjects from KSA, and study associations with CKD. Additionally, we employed multivariate analyses to examine the utility of SNPs and CKD-related biomarkers in CKD risk progression.

## Methods

Following research ethics approval from the University of Dammam Institutional Research Board (IRB), blood samples from 160 Saudi Arabian patients with CKD were collected in the outpatient Nephrology department at King Fahd University Hospital. All participants gave informed consent. To confirm disease associations, 189, ethnically-matched donors with no prior medical or family history of CKD were recruited for comparison to a healthy population. Informed consent for DNA analysis was obtained from all research participants under the supervision of the IRB.

DNA was extracted from whole blood using QIAmp Blood DNA mini kit (Qiagen, Germany). Eight polymorphisms (*SHROOM3:* rs9992101, rs17319721; *SLC7A9:* rs4805834; *MYH9:* rs4821480, rs4821481, rs2032487, rs3752462; *CST3:* rs13038305) were genotyped using TaqMan® assays (ThermoFisher, USA). Call rates greater than 95% and assessment for Hardy-Weinberg Equilibrium (HWE) were established for quality control.

Allele frequencies and genetic variant association with eGFR from European descent populations were obtained from the CKD genetics consortium (CKDGen) [13]. The CKDGen cohort is composed of including data from 67 studies and up to 133,814 participants in population-based and randomized control trials. The beta coefficients of the linear regression were corrected for age [2], sex, and population stratification in CKDGen. Kidney function was reported as sex- and age-adjusted log transformed eGFR (*n* = 133,814, overall mean = 87.12 ml/min/1.73 m2, standard deviation = 23.09), with 14% of participants having an eGFR < 60 ml/min/1.73m^2^. Vitamin D3 and Fibroblast growth factor 23 (FGF23) levels were obtained using the RECIPE ClinRep® HPLC Complete Kit (RECIPE Chemicals, GmbH, Munich, Germany) and the Human FGF-23 ELISA assay kit (Millipore, USA) respectively. Parathyroid hormone (PTH), serum phosphorus, serum calcium, creatinine, alkaline phosphatase (ALP), albumin and urine protein were assessed using the Flex® reagent cartridge on Siemens Dimension RxL chemistry system (Siemens Healthcare, GmbH, Erlangen, Germany). Based on clinical diagnostic criteria, patients were stratified by clinical comorbidities including Type 2 Diabetes (fasting glucose level ≥ 121 mg/dL) and Hypertension (systolic BP ≥ 130 mmHg and diastolic BP ≥ 85 mmHg).

Clinical and demographic variables were assessed for correlation with CKD status by using 2-sided, unpaired student’s t-test, Mann Whitney and chi-square tests. Association testing and Multivariate logistic regression and AUC analyses were performed using an R Companion for the Handbook of Biological Statistics (https://rcompanion.org/rcompanion/e_07.html).

## Results

**Fig. 1:**
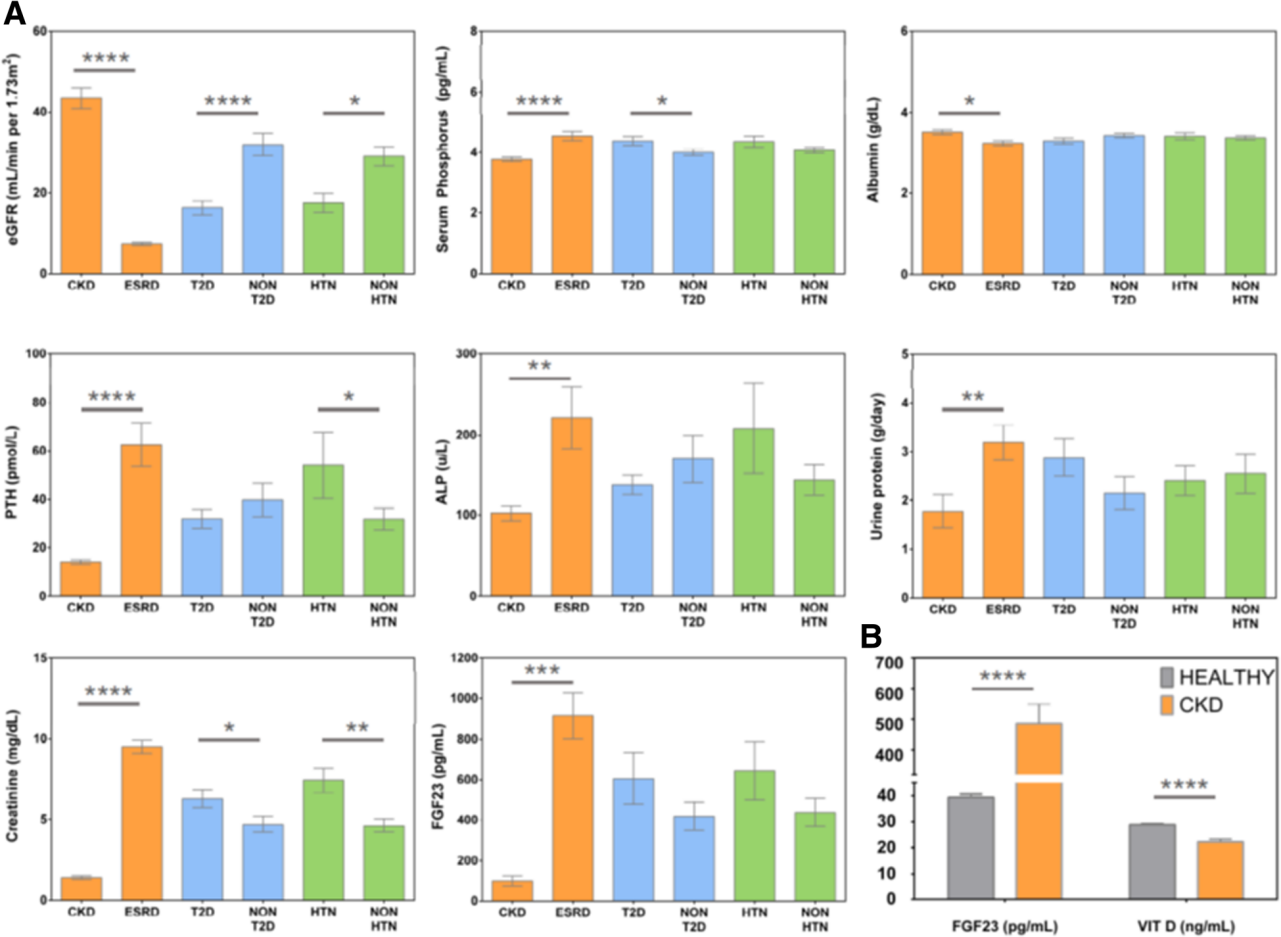
Significant associations in Saudi CKD patients were revealed in eight of ten measured biochemical analytes stratified across CKD, ESRD, Type 2 Diabetes, and hypertension. **a** One-way ANOVA analysis of clinical analytes were evaluated for statistical significance across measured CKD comorbidities. Serum calcium and vitamin D were not significant. **b** Unpaired two-tailed Student’s t-test of Vitamin D and FGF23 between healthy and CKD disease states. Significance is noted as follows: *P <  0.05, **P <  0.01, ***P <  0.001 and ****P <  0.0001 (one-way ANOVA)

The baseline characteristics of the study participants demonstrated are shown on Table 1. The median age of CKD patients and healthy donors were 47.7 years and 32.1 years, respectively, with a larger proportion of healthy donors being male (76.6% versus 53.1%, *P* < 5 × 10^− 4^). There were no significant differences in the BMI between cases and controls (mean of 27.7 and 27.9, respectively, *P* = 0.824). CKD cases included 64 patients with CKD stage 3, 20 patients with CKD stage 4, and 76 patients with CKD stage 5 (*N* = 160). Glomerulonephritis was present in 62 patients including 45 with lupus nephritis. Comorbidities in CKD patients included T2D (*n* = 57), hypertension (*n* = 37), coronary disease (*n* = 29), and congestive heart failure (*n* = 12). The mean estimated glomerular filtration rate (eGFR) in patients was 17 ml/min/1.73 m^2^ with a standard deviation of 25 ml/min/1.73 m^2^. As expected, there were significant differences between the Vitamin D3 and FGF23 levels in patients with CKD as compared to healthy donors (Fig. 1b), 22.3 ng/mL vs 28.9 ng/mL and 485.6 pg/mL vs 39.4 pg/mL (*P* < 5 × 10^− 4^), respectively. CKD patients were then stratified on the basis of one or more comorbid conditions, including hypertension and T2D, and with hypertension and diabetic nephropathy, and evaluated for significant associations to biochemical laboratory test values. Patients with end-stage renal disease (ESRD) demonstrated significantly altered biochemical laboratory values compared to those with earlier stages of CKD (Fig. 1), although no significant differences in vitamin D3 or serum calcium levels were observed (data not shown). Comparatively, patients with T2D and hypertension had significantly higher creatinine and FGF23 values as compared to those patients with CKD but without said comorbidities.

**Table 1:** Baseline characteristics

	CKD Cases	Control	*P*
N of subjects	160	189	
Age (years)	47.7 ± 17.3	32.1 ± 10.5	< 0.0005*
Sex M (%)	85 (53%)	145 (77%)	< 0.0005^¥^
BMI (kg/m^2^)	27.7 ± 6.7	27.9 ± 8.8	0.824*
Vitamin D3 (ng/ml)	22.3 ± 12.7	28.9 ± 6.4	< 0.0005*
FGF23 (pg/ml)	485.6 ± 802.6	39.4 ± 14.9	< 0.0005*
T2D (%)	57 (35.6%)	8 (4.2%)	0.0001^¥^
HTN (%)	37 (23.1%)	11 (5.8%)	0.0001^¥^

^¥^two-tailed Fisher’s exact test; * Students t test

Genotyping and quality controlled was performed in the 349 study participants of Saudi descent. All eight variants in the four tested genes were observed in appreciable frequencies in Saudis (see Table 2), along with those observed in CKDGen reference populations. Among the eight SNPs genotyped, rs4821480, a SNP mapping to the MYH9-APOL1 locus was most strongly associated with presence of CKD stage 3 or worse (OR = 1.69, 95% CI 1.22–2.36, *P* = 0.002). Linkage disequilibrium (LD) was observed between genotyped variants located in the same genes (*r* > 0.9). The direction of effects of the lead SNP from each gene were the same as those reported from the largest published cohort of European descent including 133,780 participants.

**Table 2:** Prevalence and effect of CKD risk alleles in Saudi Arabians and CKDGen

		Saudis			CKDGen		
Gene	SNP	Freq	OR (95% CI)	P	Freq	Beta	P
*SHROOM3*	rs9992101	0.26	0.89 (0.64–1.24)	0.5	0.42	−0.011	7.1 × 10^−32^
	rs17319721	0.26	0.78 (0.56–1.12)	0.14	0.42	−0.011	1.3 × 10^−37^
*SLC7A9*	rs4805834	0.16	1.07 (0.72–1.61)	0.7	0.15	0.0064	2.0 × 10^−7^
*MYH9*	rs4821480	0.24	1.69 (1.22–2.36)	0.002		N/A	
	rs4821481	0.25	0.87 (0.62–1.22)	0.43		N/A	
	rs2032487	0.25	0.87 (0.62–1.22)	0.43		N/A	
	rs3752462	0.25	0.96 (0.72–1.30)	0.8	0.33	−0.0007	9.2 × 10^−4^
*CST3*	rs13038305	0.46	1.42 (0.94–2.12)	0.09	0.26	0.0011	0.05

Odds ratio (OR) is used to quantify the presence of analyzed genes (column 1) with each population (Saudi, CKDGen). Beta is the standardized regression coefficient from CKDGen

A multivariate model was built using clinical demographic information including age and gender, which demonstrated notably correlation with CKD disease status (AUC =0.80). To further strengthen regression analysis, biochemical analyte values for FGF23 and Vitamin D3, and *MYH9* SNPs, rs4821480 and rs4821481 were tested in their capacity to enhance regression score. While FGF23 plus vitamin D levels improved association values, the combinatory enhancement of both biochemical analytes and MYH9 rs4821480 and rs4821481 genotypes generated the greatest predicative capacity and served as the best statistical model (AUC = 0.93, 95% CI 0.90–0.95, *P* <  0.0001; Table 3). Using all ten of the above described biochemical analytes across patient populations stratified for end stage renal disease (ESRD), type 2 diabetes (T2D) and hypertension (HTN) a number of significant findings were observed (Additional file 1: Table S1). Similarly, when stratifying patient population by CKD, T2D and hypertension status significant associations were found (Additional file 1: Table S2).

**Table 3:** Multivariable logistic regression analysis to evaluate improvement in CKD stage classification provided by CKD risk alleles

Model	Variables	AUC (95% CI)	*P*	Difference in AUC versus Model 1	P versus Model 1
Model 1 (Clinical)	Age & Gender	0.80 (0.75–0.84)	< 0.0001		
Model 2 (Clinical + Biochemical)	Age & Gender + FGF23 + Vitamin D3	0.92 (0.89–0.95)	< 0.0001	0.12	0.0003
Model 3 (Clinical + Biochemical + Genetic)	Age & Gender + FGF23 + Vitamin D3 + rs4821480 + rs4821481	0.93 (0.90 to 0.95)	< 0.0001	0.13	0.0001
Model 4 (Clinical + Biochemical + Genetic)	Age & Gender + FGF23 + Vitamin D3 + rs4821480	0.93 (0.89 to 0.95)	< 0.0001	0.13	0.0001

## Discussion

We tested the prevalence and CKD risk associated with alleles identified in European populations in Saudi patients. All eight variants were present in Saudi populations at appreciable frequencies, and one variant in *MYH9* was statistically significantly associated with CKD in our Saudi study population. While the remainder of alleles were not associated at statistically significant levels, our study is limited in power due to our relatively modest sample size. We did however demonstrate direction of effects that were concordant with previous reports from the larger CKDGen consortium. Genetic variants in *MYH9* are associated with non-diabetic chronic kidney disease, FSGS and HIV nephropathy, however whether the association is due to a functional variant in *MYH9* or due to LD with nearby *Apolipoprotein L1* (*APOL1*) remains a point of controversy [23]. Functional variants in *APOL1* are found on two different *G1* and *G2* haplotypes that are common in African populations (10–25%), but not observed to date in other populations [24, 25]. The function of the *G1* and *G2 APOL1* haplotypes have been recapitulated in a recently produced transgenic mouse strain [26]. It remains possible that both *MYH9* and *APOL1* are important in CKD progression, and multiethnic populations and model organisms will play key roles in untangling their relationships with CKD.

GWAS have predominantly been performed in participants of European descent, and discovery and replication cohorts in populations from around the world are needed to further enhance our understanding of complex genetic traits. The generally held belief is that a functional risk allele should exhibit the same direction of effect in all populations. However, the genetic variant tested is often not the responsible functional variant but simply associated with, termed in LD with, the truly responsible variant. As such, ethnic- and population-based differences in regional linkage disequilibrium can create associations with opposite directions of effect, especially if the genetic distance between the marker and the responsible variant is great. Moreover, as the effect is often small, genetic drift and random chance allows the minor allele frequency to vary greatly between populations, resulting in significant differences in the power to detect associations. Novel, or de novo*,* mutations may result in private mutations that are only found in specific populations. Disease prevalence can vary between populations, as observed with pediatric nephrotic syndromes in Saudis, which is likely a result of the prevalence of underlying genetic risk factors. Finally, differences in environment and genetic background produce the possibility of gene-environment and gene-gene interactions.

Over 50 loci have now been implicated in CKD progression [13] and a larger sample will be required to obtain adequate power to confidently test in Saudi populations, and overcome the multiple testing required as the number of known CKD loci grows. Large collaborative efforts such as the Saudi 100,000 genomes project [27] will facilitate larger aggregations of CKD risk loci in larger number of individuals which will greatly improve CKD risk stratification of individuals. When we combined variants in our most associated CKD locus with known CKD biochemical markers we observed a very significant AUC value (0.93). As better Saudi specific CKD genetic risk loci become available, along with more powerful CKD disease progression biomarkers, we may be able to prioritize which individuals may be suitable for interventions such as the renoprotective agents, Nisoldipine and Lisinopril. Indeed, specific targeting of specific modifiable risk factors such as hypertension and T2D, may ameliorate the majority of the CKD genetic risk loci in some subsets of individuals.

## Conclusions

In conclusion, we observed a significant association between variants in *MYH9* and CKD in Saudi Arabia, and use of multivariable logistic regression analysis showed a strong statistically significant improvement in predicting CKD using FGF23, Vitamin D and MYH9 variants. Our power to replicate previously reported associations in three other loci was limited, but we identified appreciable allele frequencies and identical direction of effect to previous reports. Further evaluation of CKD risk alleles in Saudi populations has the potential to increase our understanding of the pathogenic basis of CKD.

## Additional file

Additional file 1: **Table S1.** One-way ANOVA comparing the various Biochemical parameters in patients stratified for ESRD, T2D and HTN. **Table S2.** Correlation of biochemical parameters stratified by CKD, Type 2 Diabetes and Hypertension status. (DOCX 21 kb)

## References

[1] Baumeister SE (2010). Effect of chronic kidney disease and comorbid conditions on health care costs: a 10-year observational study in a general population. Am J Nephrol.

[2] Hoerger TJ (2015). The future burden of CKD in the United States: a simulation model for the CDC CKD initiative. Am J Kidney Dis.

[3] Alsuwaida AO (2010). Epidemiology of chronic kidney disease in the Kingdom of Saudi Arabia (SEEK-Saudi investigators) - a pilot study. Saudi J Kidney Dis Transpl.

[4] Shaheen FA, Basri NA (2002). Pre-end stage chronic renal failure: the Jeddah kidney center experience. Saudi J Kidney Dis Transpl.

[5] Köttgen A (2010). New loci associated with kidney function and chronic kidney disease. Nat Genet.

[6] Vehaskari VM (2011). Genetics and CKD. Adv Chronic Kidney Dis.

[7] Ingsathit A (2010). Prevalence and risk factors of chronic kidney disease in the Thai adult population: Thai SEEK study. Nephrol Dial Transplant.

[8] Nugent RA, Fathima SF, Feigl AB, Chyung D (2011). The burden of chronic kidney disease on developing nations: a 21st century challenge in global health. Nephron Clin Pract.

[9] Kari JA (2012). Pediatric renal diseases in the Kingdom of Saudi Arabia. World J Pediatr.

[10] MacArthur J (2017). The new NHGRI-EBI catalog of published genome-wide association studies (GWAS catalog). Nucleic Acids Res.

[11] Kottgen A (2009). Multiple loci associated with indices of renal function and chronic kidney disease. Nat Genet.

[12] Chambers JC (2010). Genetic loci influencing kidney function and chronic kidney disease. Nat Genet.

[13] Pattaro C (2016). Genetic associations at 53 loci highlight cell types and biological pathways relevant for kidney function. Nat Commun.

[14] Gorski M (2015). Genome-wide association study of kidney function decline in individuals of European descent. Kidney Int.

[15] Nishimura T, Takeichi M (2008). Shroom3-mediated recruitment of rho kinases to the apical cell junctions regulates epithelial and neuroepithelial planar remodeling. Development.

[16] Yeo NC (2015). Shroom3 contributes to the maintenance of the glomerular filtration barrier integrity. Genome Res.

[17] Menon MC (2015). Intronic locus determines SHROOM3 expression and potentiates renal allograft fibrosis. J Clin Invest.

[18] Freedman BI (2009). Polymorphisms in the non-muscle myosin heavy chain 9 gene (MYH9) are strongly associated with end-stage renal disease historically attributed to hypertension in African Americans. Kidney Int.

[19] Behar DM (2010). African ancestry allelic variation at the MYH9 gene contributes to increased susceptibility to non-diabetic end-stage kidney disease in Hispanic Americans. Hum Mol Genet.

[20] Mattoo A, Goldfarb DS (2008). Cystinuria. Semin Nephrol.

[21] Boger CA, Heid IM (2011). Chronic kidney disease: novel insights from genome-wide association studies. Kidney Blood Press Res.

[22] O'Seaghdha CM (2014). Association of a cystatin C gene variant with cystatin C levels, CKD, and risk of incident cardiovascular disease and mortality. Am J Kidney Dis.

[23] Colares VS (2014). MYH9 and APOL1 gene polymorphisms and the risk of CKD in patients with lupus nephritis from an admixture population. PLoS One.

[24] Ko WY (2013). Identifying Darwinian selection acting on different human APOL1 variants among diverse African populations. Am J Hum Genet.

[25] Bajaj A, Susztak K, Damrauer SM (2017). APOL1 and cardiovascular disease: a story in evolution. Arterioscler Thromb Vasc Biol.

[26] Beckerman P (2017). Transgenic expression of human APOL1 risk variants in podocytes induces kidney disease in mice. Nat Med.

[27] Project Team SG (2015). The Saudi human genome program: an oasis in the desert of Arab medicine is providing clues to genetic disease. IEEE Pulse.

