# Supplementary material for: Assessing known chronic kidney disease associated genetic variants in Saudi Arabian populations
Source: BMC Nephrol. 2018 Apr 17;19:88. doi: 10.1186/s12882-018-0890-9 (PMC5905143; doi:10.1186/s12882-018-0890-9)
Supplement: Supplementary file 1 — Table S1. One-way ANOVA comparing the various Biochemical parameters in patients stratified for ESRD, T2D and HTN. Table S2. Correlation of biochemical parameters stratified by CKD, Type 2 Diabetes and Hypertension status. (DOCX 21 kb) [file 12882_2018_890_MOESM1_ESM.docx]

**SUPPLEMENTARY DATA for Cyrus *et al.* Assessing Genetic Variants Associated with Chronic Kidney Disease Risk in Saudi Arabia**

Additional file 1: Table S1 – One-way ANOVA comparing the various Biochemical parameters in patients stratified for ESRD, T2D and HTN

|  | CKD vs. ESRD | | | |
| --- | --- | --- | --- | --- |
|  | **CKD**  **Mean ± SD** | **ESRD**  **Mean ± SD** | **F** | **p** |
| EGFR ML/min 1.73 m2 | 43.44±23.96 | 7.42±3.2 | 168.936 | 0.0001 |
| Serum phosphorus (pg/mL) | 3.78±0.68 | 4.54±1.29 | 22.314 | 0.0001 |
| Serum Calcium (mg/dl) | 9.51±8.14 | 8.62±1.15 | 0.892 | 0.346 |
| Albumin (g/dl) | 3.51±0.54 | 3.23±0.55 | 10.541 | 0.001 |
| Creatinine (mg/dl) | 1.42±1.01 | 9.52±3.45 | 423.202 | 0.0001 |
| PTH (pmol/L) | 13.96±7.93 | 62.58±78.36 | 31.997 | 0.0001 |
| Vitamin D3 (ng/mL) | 21.53±13.17 | 23.18±12.06 | 0.678 | 0.411 |
| ALP (u/L) | 103.10±88.38 | 222.16±336.32 | 9.786 | 0.002 |
| Urine protein (g/day) | 1.78±2.91 | 3.19±2.76 | 9.836 | 0.002 |
| FGF23 (pg/ml) | 97.45 ± 220.49 | 914.69 ± 977.98 | 55.570 | 0.0001 |

|  | CKD with HTN vs. CKD without HTN | | | |
| --- | --- | --- | --- | --- |
|  | **HTN**  **Mean ± SD** | **Non HTN**  **Mean ± SD** | **F** | **p** |
| EGFR ML/min 1.73 m2 | 17.45±14.86 | 29.18±26.96 | 6.400 | 0.012 |
| Serum phosphorus (pg/mL) | 4.36±1.10 | 4.07±1.08 | 261.727 | 0.000 |
| Serum Calcium (mg/dl) | 8.8±1.23 | 9.17±6.76 | 0.109 | 0.741 |
| Albumin (g/dl) | 3.41±0.52 | 3.37±0.57 | 0.146 | 0.703 |
| Creatinine (mg/dl) | 7.42±4.64 | 4.62±4.62 | 10.427 | 0.002 |
| PTH (pmol/L) | 54.10±83.23 | 31.937±49.32 | 4.045 | 0.046 |
| Vitamin D3 (ng/mL) | 21.70±13.37 | 22.50±12.47 | 0.113 | 0.737 |
| ALP (u/L) | 209.58±345.18 | 145.21±209.62 | 1.930 | 0.167 |
| Urine protein (g/day) | 2.55±2.06 | 2.40±3.10 | 0.076 | 0.783 |
| FGF23 (pg/ml) | 643.87 ± 878.82 | 438.04 ± 775.70 | 1.881 | 0.172 |

|  | CKD with T2D vs. CKD without T2d | | | |
| --- | --- | --- | --- | --- |
|  | **T2D**  **Mean ± SD** | **Non T2D**  **Mean ± SD** | **F** | **p** |
| EGFR ML/min 1.73 m2 | 16.25±13.67 | 32.15±28.16 | 16.045 | 0.0001 |
| Serum phosphorus (pg/mL) | 4.38±1.07 | 4.00±1.08 | 4.573 | 0.034 |
| Serum Calcium (mg/dl) | 10.08±9.88 | 8.53±0.87 | 2.513 | 0.115 |
| Albumin (g/dl) | 3.29±0.56 | 3.42±0.56 | 1.977 | 0.162 |
| Creatinine (mg/dl) | 6.30±4.01 | 4.69±5.05 | 4.292 | 0.040 |
| PTH (pmol/L) | 32.04±29.02 | 39.83±70.76 | 0.631 | 0.428 |
| Vitamin D3 (ng/mL) | 20.77±10.04 | 23.17±13.85 | 1.325 | 0.252 |
| ALP (u/L) | 138.26±90.10 | 172.35±302.54 | 0.688 | 0.408 |
| Urine protein (g/day) | 2.88±2.76 | 2.15±3.00 | 2.298 | 0.132 |
| FGF23 (pg/ml) | 605.67 ± 957.38 | 419.21 ± 698.83 | 1.993 | 0.160 |

# Table S2 – Correlation of biochemical parameters stratified by CKD, Type 2 Diabetes and Hypertension status

| **Parameters** | **non-ESRD (N) vs ESRD (N)** | **non-T2D (N) vs T2D (N)** | **non-HTN (N) vs HTN (N)** |
| --- | --- | --- | --- |
| FGF23 | <0.0001 | 0.0264 | 0.0115 |
| PTH | <0.0001 | 0.1194 | 0.2597 |
| Phosphate | <0.0001 | 0.0051 | 0.1710 |
| Calcium | 0.5269 | 0.0307 | 0.1376 |
| Creatinine | <0.0001 | 0.0003 | 0.0002 |
| ALP | <0.0001 | 0.1186 | 0.1140 |
| Vitamin D3 | 0.1557 | 0.6729 | 0.6344 |
| Albumin | 0.0027 | 0.0976 | 0.9871 |
| Urine protein | <0.0001 | 0.0133 | 0.1367 |
